# Abandoned in No Man's Land: A Qualitative Study on Patient Experiences While Waiting for Elective Coronary Artery Bypass Surgery

**DOI:** 10.1155/nrp/3139277

**Published:** 2025-09-02

**Authors:** Dorte Baek Olsen, Ida Elisabeth Hoejskov, Malene Missel

**Affiliations:** ^1^Department of Cardiothoracic Surgery, Rigshospitalet, Copenhagen University Hospital, Copenhagen, Denmark; ^2^IMPACT-Research & Care, Rigshospitalet, Copenhagen University Hospital, Copenhagen, Denmark; ^3^Department of Heart Disease, Rigshospitalet, Copenhagen University Hospital, Copenhagen, Denmark; ^4^Faculty of Health and Medical Sciences, University of Copenhagen, Copenhagen, Denmark

**Keywords:** adult, CABG surgery, hermeneutic, ischemic heart disease, patient experience, phenomenology, prehabilitation, qualitative, waiting

## Abstract

Coronary artery bypass grafting is the primary treatment for patients with ischemic heart disease and multivessel disease to improve survival, symptoms, and quality of life. However, patients waiting for coronary artery bypass grafting surgery often struggle with advanced age, frailty, and inactivity, leading to anxiety and uncertainty. Psychological distress, pain, and sedentary behavior can worsen their condition. Prehabilitation, combining exercise, education, and support, aims to improve readiness for surgery, but its success depends on understanding patients' perspectives, while this study explores the experiences of patients awaiting coronary artery bypass grafting surgery to contribute insights into the design of future prehabilitation programs. This qualitative study aimed to explore the phenomenon of waiting to have elective coronary artery bypass grafting surgery based on patients' lived experiences. The study used a hermeneutic–phenomenological approach and conducted purposely selected in-depth interviews with one female and nine men the day before surgery. Analysis of the data revealed themes based on experiences of being abandoned in no man's land, balancing between feeling imprisoned and hoping to escape, preparing to fight, and feeling weak and dependent on social support. The phenomena this study uncovered underscore the complexities inherent to the lived experience of waiting for coronary artery bypass grafting surgery. Understanding and integrating these aspects when planning prehabilitation programs for patients waiting for coronary artery bypass grafting surgery have substantial clinical relevance, particularly for enhancing healthcare practice when developing targeted interventions.

## 1. Introduction

Coronary artery bypass grafting (CABG) is the primary treatment for patients with ischemic heart disease (IHD) and multivessel disease. However, patients waiting for CABG surgery often struggle with advanced age, frailty, and inactivity, leading to anxiety and uncertainty. Prehabilitation, combining exercise, education, and support, aims to improve readiness for surgery, but its success depends on understanding patients' perspectives, while this study explores the experiences of patients awaiting CABG surgery to contribute insights into the design of future prehabilitation programs.

## 2. Background

IHD accounts for more than four million deaths annually in Europe, placing a great strain on healthcare resources [1, 2]. Revascularization with CABG is the gold standard treatment in patients with multivessel disease for relieving symptoms of angina and improving exercise capacity, quality of life, and survival rates [3, 4]. However, patients awaiting CABG surgery often face challenges such as advanced age, frailty, comorbidity, and physical inactivity [5, 6]. Moreover, studies confirm that the time spent waiting for CABG surgery is a period of great uncertainty that appears to heighten anxiety regarding physical activity, mainly because of the current cardiac conditions or diagnosis, such as distress and pain [7–11]. Evidence shows that patients deteriorate functionally and psychologically during this period prior to surgery [6, 7]. Findings indicate [6, 9] that patients scheduled for elective CABG surgery often wait at home and undergo little or no physical training or other interventions prior to the procedure, generally because they are concerned about the surgery, potential complications, and physical limitations. While a decrease in physical activity quickly leads to a decline in physical fitness and reduces the physiological capacity to withstand the side effects of surgery [7, 12], innovative strategies between diagnosis and surgery are required to alleviate the risk of adverse outcomes for these patients [13]. Prehabilitation, a proactive process aimed at enhancing functional capacity and psychological well-being to withstand the upcoming physiologic stress of surgery and, consequently, to avoid complications [14–18], involves a combination of exercise, training, education, and social support. As such, prehabilitation represents an opportunity for patients waiting for CABG surgery to participate in their preoperative care by preparing themselves for surgery. Although the benefits of prehabilitation are known [19, 20], existing research has not fully explored how the waiting period before surgery affects patients' daily lives or how well they are able to prepare. Moreover, current prehabilitation programs may lack personalization and fail to consider individual needs [21–23]. Factors such as the nature of illness, patient characteristics, and their sense of time can all shape how they experience this waiting period [9, 24]. Despite this, patient experiences, while waiting for CABG surgery at home, remain underexplored even though the lived experience during this period may have a significant impact when planning future prehabilitation programs [21, 22], ensuring that interventions are meaningful and appropriate to promote their effectiveness [25]. Thus, understanding what is at stake for these patients in the waiting period is urgently required to create optimal conditions and person-centered interventions for activities related to prehabilitation. This study sheds light on the home experiences of patients waiting for CABG surgery to contribute crucial insights into the design and theoretical underpinnings of future prehabilitation programs.

## 3. The Study

### 3.1. Aim

The aim of this study is to explore the phenomenon of waiting to have elective CABG surgery based on patients lived experience.

## 4. Methods

### 4.1. Design

Inspired by Max van Manen's [26, 27] hermeneutic–phenomenological methodology, this study works to illuminate the unique structure of the human experience by analyzing experiences as they are lived [27, 28]. To explore the phenomenon of waiting for CABG surgery, we employed a phenomenology of practice as a human science research methodology [26]. When applying this methodology, which is well-suited for studying practitioners who may lack awareness regarding the subtleties of other people's experiences in their day-to-day practices, researchers describe and interpret the lived world as experienced in everyday situations [29]. Patients who must wait for surgery while living with cardiac disease face an extraordinary life situation, which is why phenomenological insight into human existence is of interest and relevance [30]. We were guided by van Manen's four lifeworld existentials, lived time, lived space, lived body, and lived human relations, as our methodological framework to conduct qualitative patient interviews and for the subsequent data analysis [26, 27]. In this study, interpretation unfolded through a cyclical process of reading, reflective writing, and thematic analysis, wherein we moved between individual accounts and the emerging whole to uncover existential meanings. This process was not aimed at producing objective truths but at illuminating the significance of “waiting” as experienced by participants within their lifeworld. Reflexivity was central to this interpretive stance. We engaged in ongoing reflection and journaling to remain aware of our own preunderstandings and how these shaped the interpretive process. This dialogical engagement with the text ensured that interpretation remained grounded in participants' voices while being enriched by thoughtful, situated analysis. The two authors (D.B.O. and I.E.H.) have extensive experience in cardiac surgical care and (M.M. and I.E.H.) have extensive experience in previous phenomenological studies. The Standards for Reporting Qualitative Research (SRQR) [31] was followed to ensure rigor in the study (Supporting Information [available here]).

### 4.2. Participants

Participants were purposely selected in a heart surgery ward at a university hospital, where approximately 400 patients with IHD await CABG surgery annually. The inclusion criteria were ≥ 18 years of age, diagnosed with IHD, and scheduled for CABG surgery, in addition to the ability to speak and understand Danish. Recruitment took place on the day of admission, the day before surgery, after participants had spent at least 2 weeks waiting at home. They got as much time as they needed to consider participating in the interview. To ensure diverse perspectives, participants were purposively sampled based on a range of sociodemographic and clinical factors including sex, age, marital status, employment status, and place of residence. Ten patients participated in the study from September 2020 to May 2021. The present study involved a total of 10 semistructured interviews with the participants comprising one woman (10%) and nine men (90%) aged between 44 and 83 years (mean: 67.4 years) and had waited 9–90 (mean: 41.6) days for their surgery. Two participants were still in the labor market, eight were retired, four lived alone, and six were married. The majority of participants were men, which reflects the gender distribution of patients undergoing CABG surgery.

### 4.3. Data Collection

We conducted 10 individual in-depth interviews when patients were admitted the day before their elective surgery. These interviews aimed to explore and gather personal accounts, narratives, and anecdotes that illuminate the experience of waiting for CABG surgery. The interviews took place in a quiet room on the heart surgery ward. The interviews were conducted face-to-face without anyone else present besides the participant. A semistructured interview guide inspired by Max van Manen's four lived lifeworld existentials was used. The interview questions were designed to encourage patients to share their experiences openly and comfortably with the researcher, allowing them the freedom to narrate their story in their own way. Given the importance of intersubjectivity in qualitative research, a phenomenological approach was employed to foster attentiveness [26] that involved prioritizing eye contact between the participant and the researcher, creating a sense of trust, and listening carefully to the participants' narratives. Interviews were conducted by the first author, an experienced female qualitative researcher who also worked as a clinical nurse specialist but was not involved in the clinical care of the included patients. Each interview began with a broad opening question, such as “Could you please tell me about your experiences with waiting for surgery for your heart disease while at home?” Narrative accounts of patient experiences were encouraged, and questions such as “How did you feel about getting a heart disease diagnosis?” and “How did you feel about being referred to and preparing for surgery?” were also asked, and probing questions such as please tell me more “and” please tell me more about how you felt (related to body, space, time and relation) in that situation were used. Participants were interviewed once from September 2020 to May 2021. The interviews were audio recorded and lasted 12–42 min, and the first author transcribed each interview verbatim. The interviews vary in duration and underscore differences in how individuals articulate their experiences and narratives. The researchers adopted a phenomenological attitude while conducting the interviews to identify the meaning of the phenomenon “waiting for” as it presents itself in and as human experience [27, 30, 32].

### 4.4. Data Analysis

Van Manen's [26, 27] analytical steps combined with a holistic, selective, and detailed reading of the interviews guided the analytical exploration of the participants' experiences of waiting at home for their CABG surgery [26]. The analysis aimed to uncover the prereflexive descriptions the participants gave to reach a deeper level of collective meaning. Analyses of the interviews were carried out by D.B.O. and M.M.. During this process, we worked to remain focused on the study's purpose and to hold presuppositions, ideas, and feelings abbey during the various analytical phases. We thoroughly analyzed each interview to identify patterns and themes capturing patient experiences when waiting for cardiac surgery in an interpretive process involving a detailed, line-by-line examination of the interview transcripts based on van Manen's analytical framework [26, 27]. Throughout this process, we meticulously searched for descriptions that vividly conveyed what it is like to be a person with heart disease while waiting for heart surgery at home. The textual analysis was structured to maintain a focus on the core research question while drawing up a coherent thematic narrative. In the initial stage, the interviews were approached with an open mind to grasp the underlying real-life experiences [26, 27] The text was then segmented into clusters of meaning that were continuously reviewed in relation to the entire text to enhance overall comprehension. Thus, the challenge was to find and “bring to the surface from the depths of the ocean of life” [26] quotes illustrating specific experiences in a powerfully evocative way [28]. Finally, these clusters were synthesized into essential themes encapsulating the central phenomenon of waiting for CABG surgery at home. Each theme provides a comprehensive portrayal of intricate life experiences, aiming to reveal the underlying structure of meanings. We employed existential reflections to explore lived experience with lived time (temporality), lived space (spatiality), lived body (corporeality), and lived human relations (relationality). Linguistic tracing involved paying attention to the conceptual and etymological aspects of the language used when waiting for CABG surgery. In our analysis, van Manen's four existentials, lived space, lived body, lived time, and lived human relation, served as a reflective lens through which we interpreted participants' narratives. While we did not organize the results into separate sections for each existential, these dimensions informed our thematic interpretation. For example, themes related to participants' sense of waiting and anticipation were interpreted through the lens of lived time, while descriptions of bodily vulnerability and physical sensations were understood in relation to the lived body. Similarly, experiences of isolation or connection with others were considered through lived relation.

#### 4.4.1. Rigor and Reflexivity

We employed a phenomenology of practice as a human science research methodology [26] to explore the phenomenon of waiting for CABG surgery. Data collection and analysis were discussed among the researchers during the entire study. The researchers remained open towards the phenomenon of interest from the first salient thoughts all throughout collecting, analyzing, and disseminating the data and the writing process. Staying open in a second-person perspective was, however, challenging since our natural attitude (prejudgments) continuously evades the mind [33]. We tried to incorporate an attitude in which “seeing” the others' experience was gradually instilled into us in reading, discussion, and writing processes. We were alternately dwelling in and distancing from data, thus making the content subject to scrutiny through reflection and mutual discussion.

#### 4.4.2. Theoretical Inspiration

Integrating our findings with the existing literature offered novel insights into the experience of waiting for CABG surgery. In the Discussion section we applied theoretical frameworks such as van Manen's phenomenology [26–30], Meleis' transition theory [34], Antonovsky' s [35] sense of coherence (SOC) theory, and Charmaz's theory [36] on chronic illness to provide a deeper understanding of how patients perceive and navigate their experiences. Utilizing van Manen's phenomenological methodology, this study meticulously explores and interprets the experiences of patients awaiting elective CABG surgery. By synthesizing our findings within this broader theoretical and clinical context, the discussion section aims to elucidate the multifaceted nature of patient experiences.

### 4.5. Ethical Considerations

This study was approved by the Danish Data Protection Agency (P-2020-724). The study was conducted in accordance with the ethical principles outlined in the Declaration of Helsinki [37]. The participants received oral and written information about the study's purpose and their right to withdraw from the study at any time without consequences for their treatment and were told that interview data would be treated confidentially. Data were pseudoanonymized using identification codes.

## 5. Findings

### 5.1. Themes

The findings of this study shed light on the lived meanings the individual's experienced waiting for elective CABG surgery. The findings are based on experiences of being abandoned in no man's land, balancing between feeling imprisoned and hoping to escape, preparing to fight, and feeling weak and dependent on social support. These themes define the existential state patients are in when waiting for cardiac surgery, which will be described and unfolded to explicate the meaning of the phenomenon “waiting for” as it presents itself in and as the participants' experience. Quotations will be used as examples of this explicated meaning, and some degree of overlap is inevitable, as experiences and expressions may carry multiple meanings.

#### 5.1.1. Abandoned in No Man's Land

One of the primary experiential aspects of waiting for CABG surgery was that participants felt they were being abandoned and left to fend for themselves in no man's land. When discharged after an acute cardiac event and told to take it easy at home to wait for their surgery, the participants felt isolated and alone and had no idea what the surgery involved or what would happen to their hearts. The theme of being abandoned in no man's land represents the feeling participants had of being thrown into unknown, unclaimed, or uninhabited territory, where they had to manage on their own without previous experience to draw on. In this unknown landscape, thoughts and concerns arose about life, death, treatment, and the future, causing patients to face specific existential uncertainties while waiting for surgery at home as this man described:

“Then you get a call about what the situation is and are told that the best thing for you would be a bypass, and if you agree, you have to show up on such and such a date. Then you go there, and you don't know much” (p9).

The participants find it distressing when the hospital tells them that bypass surgery is necessary. Some describe it as being knocked out and say that they are affected existentially as they struggle to deal with their thoughts and uncertainties in daily life. Some experience troubling physical symptoms that resemble the signs of heart disease they are familiar with. The perception that the body is using a different, yet similar language creates confusion about how to interpret the body's signals, as narrated by this woman:

“Then you're suddenly told that you must have a bypass, which hits you like a ton of bricks and is too much to digest at once. It has deeply affected me psychologically, and I tell myself to block my thoughts. Every time I thought about it or talked about it, I nearly ended up with almost all the symptoms. It has been a huge psychological burden” (p7).

Embodied experiences that involve being confronted with one's own mortality can lead to racing thoughts and sleeplessness, gravely interrupting the daily lives of participants and experienced as particularly difficult to deal with alone, as described by this man:

“A doctor came in and it scared me. I can't shake it off (crying) … he came in and said: hello … (showed me how they crack open the chest) Haven't you been told, he said? It (the fright) has not left me since (crying). My older brother has had one or two bypasses. Yes, I've been scared. At night when I go to sleep, it spins around (points to his head), forcing me to get up and watch TV until I'm so tired I fall asleep” (p8).

Even though it is a difficult period, the participants talk about how they and their relatives have taken responsibility for assessing what is possible to do in daily life while waiting. They have listened carefully to the healthcare experts; however, they view the information they received as superficial and lacking in specific personalized guidance. Some were told to take it easy, without being informed exactly what this entails, told by this man as:

“I've been told (after coronary angiography) that I can do what I normally do. I shouldn't run a marathon or overexert myself” (p6).

During the waiting period, participants thus had to judge for themselves what and how much physical exertion they could handle. They talk about the feeling of being left to themselves to assess their body's limitations and possibilities. Compounding the feeling of being alone in an unfamiliar territory is their concern for their relatives due to a lack of knowledge about the surgery, as described by this man:

“It's been hard for my loved ones, so I could have used a conversation earlier in the process that explained the surgery to us. I've been pretty much on my own with it. I think we could have used information about how much you can lift and stuff like that, you see?” (p9).

Feelings of uncertainty and questions about whether the participant wishes to have surgery are also components of what they experience while waiting. Participants talk about the doubts they have about the surgery in terms of how it will affect their lives and whether undergoing surgery is the right decision. They mention feeling forlorn and unequipped to deal with these considerations on their own and talk about having a strong desire to discuss various issues with a healthcare professional, as this man told:

“Then I received a letter stating that the hospital had determined it would be best if I did the surgery in question. No one asked me (crying), and I would rather not” (p8).

Despite the uncertainty they face not knowing how long they will have to wait, some participants demonstrate resilience by building up a sense of control over their uncertain life situation by using various coping strategies. Some participants force themselves to continue their familiar everyday lives and ordinary pursuits to keep worries and stress at bay, others focus more on practical preparation, such as the distance to the hospital and parking facilities, in addition to acquiring information from acquaintances and on the internet. There are, however, also participants who let their emotional distress take over and fail to consciously suppress it. No matter what approach the participants take, they all find themselves, to some extent, in no man's land with no escape in sight. Some participants experience feeling sidelined in their own lives, pushed into the passive role of observer, and suddenly left to experience life from the outside. They mention that in this position they are not as active as they usually are and are relegated to simply waiting position, stirring feelings of powerlessness, vulnerability, and not being fully in command of one's life. Waiting in the shadows also creates a feeling of disconnectedness to the diseased part of one's own body, the heart, which is a symbol of life itself, but neither the heart nor life can be trusted any longer. Furthermore, participants comment that they presume this state will persist until the surgery has been successfully completed, as narrated by this woman:

“I think that fifty-two days is a long time. I couldn't get it done fast enough after I was told I had to have a bypass. I couldn't keep waiting and I'm glad it happened within a month because I'm not sure I could have stood it for months and had to wait like that. It was too much” (p7).

#### 5.1.2. Balancing Between Feeling Imprisoned and Hoping to Escape

Waiting for cardiac surgery is also a journey that involves balancing between possible risks and hope in terms of the future. Patients hope that the surgery will be successful but simultaneously entertain thoughts and concerns about who they will be once they recover from surgery. Patients experience moments of despair and uncertainty when balancing upsetting emotions and the hope and belief that they will recover and improve after surgery. Successfully navigating IHD requires a combination of resilience and support from relatives and healthcare professionals to foster a belief in the possibility of recovery and improvement after surgery. Unique, personal, and ongoing, this process entails striking a balance while waiting for surgery between the repercussions of the illness and hope for the future, as narrated by this man:

“It's been a bit up and down. One day you feel great and the next you think about it a little more. I've otherwise done well, also because I have a sense of clarity about what's going to happen. The alternative is that it will go wrong sooner or later” (p2).

Disturbing thoughts about illness and death are pervasive during the wait time, intruding even when participants consciously try to unwind by, for instance, watching TV to have a break from speculating about their surgery and the risk of not surviving, as told by this man:

“You think about what's going on and stuff like that … I think your mood can fluctuate a bit … it's mostly when you're just sitting and watching TV, when you want to escape and think about something … and think about what's going on. It's a big operation so you think … what now? … What if it goes wrong, what then?” (p3).

At the same time, participants talk about their dreams and hopes for the future, optimistic that the desire to get in better physical shape after surgery will improve their quality of life and put them on track to regain a meaningful life or as they state, “to get your life back.” Thus, the longing to return to their familiar everyday lives, where their body and its functions and emotions fade from their consciousness, is closely linked to their impending surgery, which represents the hope of regaining a functional body. The wait time, however, represents an existential threat. This balancing act constitutes a central temporal aspect of waiting for CABG surgery, as told by this man:

“I look forward to the time after surgery and just after recovery because I know it's a big operation and that every operation is unique. There's always a risk … but I choose to look at it as worth it. Because afterward, when if everything goes well, you're a better and stronger person and I look forward to that. Being able to get back just some of what I once had, well, that means everything” (p5).

For many participants, the wait time for CABG surgery is fraught with a complex mixture of emotions ranging from hope for a renewed life and distress about the struggle the current limitations of IHD impose on them. Although it is challenging to wait for surgery, many participants see the surgery as a turning point, as a form of liberation, and envision returning to a life, where they can actively participate in daily activities and regain independence. They describe their current state as being trapped in a prison of illness, and that they feel like they are being themselves yet are not themselves. In other words, they are struggling to recognize themselves as the person they once were. This existential limbo underscores their desire for a functional body that can engage in social activities and other meaningful pursuits, highlighting the surgery as a crucial gateway to reclaiming their sense of self and freedom, described by this man as:

“For me, the operation is a chance and a matter of whether I must spend a lifetime in a prison where I can't do a thing or whether I might be able to escape that prison. I'm in my 40s, so come on, it (life) isn't over yet, but it has been for me in recent years. To get some of that (life) back … then I can't look at the risks of the operation with anything but a smile. One way or another, I'll be free …because it (life) can't get any worse than it already is” (p5).

#### 5.1.3. Preparing to Fight

Prior to heart surgery, the participants manage everyday life waiting at home by using coping strategies to deal with a changed body due to IHD and prepare for surgery as if going into battle. Preparing to fight, however, is a lonely experience characterized by yearning for someone to reflect with on the body's limitations and possibilities. Unsure of what their bodies can handle, participants mention being forced to interpret their body's signals alone and having to slow down to find a rhythm and strategy for coping with everyday life at home. Doing so requires surplus energy and a belief in oneself that can be difficult to muster in their situation but that is essential to the ability to mobilize a readiness to fight, as described by this man:

For others, the wait time gives them the opportunity to socialize and refuel with friends and family and accomplish practical tasks prior to surgery. The various preparation strategies involve addressing profound existential issues such as identity and the potential impact of the procedure on their lives. Facing surgery may bring about a heightened sense of awareness about one's mortality and vulnerability. Recognizing that its outcome is uncertain can lead to reflecting on the fragility of life. Thus, existential preparation is a deeply personal process. While waiting for surgery, some participants find solace in meeting with friends and family as a way to deal with existential issues, while others find a sense of peace in accepting their new life situation. While waiting, several participants are concerned about whether they can actively do something to strengthen their bodies before CABG surgery, as this man told:

“For me, a month and a half has been fine because you can get things in order before you have surgery, for example, if you need to go to the dentist. There is also the issue of having a lack of mobility for a while afterward, which is why I've made sure socialize positively with friends in the evening. So, I think that it has suited me very well that I have had time to … also just to accept it (having surgery)” (p5).

During the wait time, others independently come up with their own strategies to change their lifestyles by adjusting their diet and their expectations about becoming more physically fit by exercising at home, allowing them to take greater control, possibly engaging in a transformative and empowering process. A conscious effort is required to alter habits and make lifestyle changes to live more healthily, causing participants to reflect on their current lifestyle and embrace areas ripe for change, such as eating better. While waiting for surgery, participants experience a willingness to take control of their lifestyle, which requires commitment, patience, and the motivation to embrace change by making intentional choices, as described by this man:

“If you could do some exercises at home to get a bit stronger, I would definitely do it. You spend time going back and forth to the gym, so if you could work out at home, it would be better. Right now, my goal is to do strength training again once it (surgery) is over because I've gotten a bit flabby” (p2).

Waiting for heart surgery and having strategies to prepare by completing work tasks and delegating work responsibilities represent another way some participants reduce their worries and assert control over their work lives. Embracing these activities serves to mentally prepare them before surgery in a way that is crucial to not only their physical well-being but also their mental and emotional resilience, as described by this man:

“At my job, I manage various employees, and I have numerous tasks that I have to hand over to others. The phone rings 20 times a day and I get 50 emails, so there's a lot of people I have to talk to about this (heart surgery). So, in a way, I've been busy, and I haven't been worried” (p2).

Other participants prepare by staying calm and engaging in manageable activities such as sitting quietly reading and taking one day at a time. For others, the wait time involves creating a space for themselves, for example, by taking daily walks. Physically creating the space to be alone, unwind, or engage in activities that bring joy and relaxation is a healthy strategy that establishes an emotional distance to the participants' situation that promotes well-being. Creating and allocating time for self-reflection allow the participants to better understand their thoughts and emotions. As such, waiting for surgery involves patients experimenting with activities to identify and become attuned to what individually works for them.

#### 5.1.4. Feeling Weak and Dependent on Social Support

Waiting for CABG surgery involves living in fear and being on one's own, resulting in participants experiencing themselves as weak and dependent on social support. Participants describe feeling alone and often say they do not have anyone to share their unchecked thoughts with about the surgery, including topics involving the existential issues that arise in the face of a serious illness. The participants must explain the seriousness of what they are facing with family and friends. If without support in everyday life or any recognition of how distressing their situation is, participants may experience existential loneliness, as narrated by this woman who was living alone:

“It's very much down to the raw psychology because I'm well aware that what you experience is psychological when you have to go alone with it. It was just nerves … you get turned upside down … so that's why it was a bit of a shock” (p7).

During the wait time, participants had concerns about what would happen with the body, the future, and one's identity. Furthermore, they express a need to communicate with healthcare professionals as a supportive strategy to manage difficult thoughts when feeling abandoned in no man's land. Stranded in this situation, they feel the need to share their feelings of loneliness with a healthcare professional and, thus, receive some recognition about the difficulty of their situation as human beings. Sharing difficulties with another human being can partially alleviate the burden and suffering and may be helpful, as explained by this man:

“When talking to the doctor prior to the operation, you also had a chat with a nurse about any concerns, and then there could be some kind of template for what the nurse asks about” (p5).

Of great importance to the participants during the wait time before surgery is having the ability to take care of themselves and being independent of others' help. Autonomously making decisions about their own lives and being independent are crucial to them to maintain a sense of independence and control in an uncontrollable situation. Being independent allows them to have control over their own lives when waiting for surgery and empowers them to make decisions and choices based on their own values and needs, contributing to their individual resilience and adaptability to the situation, as this elderly widower said:

“I manage on my own, and I haven't had help and don't need it [laughs] because the local authorities have tried. I don't want to talk to them. I can manage on my own and then I have my friend who lives upstairs. Everything is fine. My friend is a gym teacher, and she knows all about muscles and tendons. She's well versed in all those things and she's available to help with training” (p3).

During the wait time, the participants have supportive conversations with friends about life and death that allow them to share their fear of not surviving the upcoming surgery. At the same time, sharing their concerns about life and death appears to be difficult for various reasons, with some participants using black humor as a buffer to create distance in their friendships. The vulnerability they experience due to their emotional vicissitudes and trepidation hinders being open about the uncertainty of waiting for CABG surgery. Furthermore, some participants fear that their friends will judge or misunderstand them, which is why discussions about life and death issues can be associated with a sense of vulnerability. Consequently, some participants are reticent in terms of opening up about deeply personal matters, leaving them feeling isolated, unsupported, and unacknowledged. Some participants are preoccupied with whether their friends fully grasp the gravity of their situation to provide meaningful support. They may feel more comfortable processing their emotions alone or seeking support from healthcare professionals, as this man narrated.

“I have friends, and I have a morbid sense of humor and so do they, so that's kind of how we've talked about it. We haven't talked seriously because it's been a bit like, of course I'm going to make it [heart surgery]. The few friends who have asked if I've planned if things don't go as they should, I wouldn't say I've brushed it off with humor, but if I'm to be completely honest, I've done it because I have the attitude that either I make it or I don't” (p5).

Participants explain how the wait time is associated with uncertainty about how sick they are, how bad their heart is, and what exactly CABG surgery involves. This uncertainty creates a deep desire to speak with healthcare professionals to receive individualized guidance and support about CABG surgery and how it will affect their everyday lives in the future in the hope that this will give them peace of mind and reassurance. Uncertainty about the future and a pervasive sense of anxious moodiness amplify the existential loneliness participants experience while waiting for surgery. The lack of a guaranteed positive surgical outcome and specific knowledge about how best to prepare for surgery underscores the need for tailored and compassionate communication from healthcare professionals. Being recognized as a unique individual with specific emotional and informational needs is crucial for participants to help alleviate the intense emotions associated with the wait while also building trust and providing a sense of comfort in the face of life-altering surgery.

## 6. Discussion

This study has unveiled lived meanings experienced by individuals waiting for elective CABG surgery, illuminating thematic aspects of their existence. Our findings introduce perspectives on the experiences of waiting, particularly by emphasizing the existential aspects of the wait time, as the participants' lived experience unfolded through four interwoven existential themes: being abandoned in no man's land, balancing between feeling imprisoned and hoping to escape, preparing to fight, and feeling weak and dependent on social support. These findings underscore that waiting for cardiac surgery is not only a shared emotional and temporal experience but also a deeply interpretive one. While patients report similar physical and psychological challenges, the meanings they assign to these experiences differ significantly. This interpretive variability reflects the hermeneutic orientation of the study, highlighting how individuals make sense of their situation considering personal, relational, and existential contexts. In the following discussion, the themes will be explored and contrasted considering existing research, as well as interpreted through theoretical perspectives inspired by Max van Manen, Meleis, Charmaz, and Antonovsky. Unlike previous studies [9, 38–41], our research highlights the patients' profound feeling of being abandoned in no man's land, where the participants experience a lack of direction and overpowering uncertainty about their lives during the wait. This critical insight into the existential dimension of waiting represents a key contribution to the existing literature, underscoring the deep challenges patients face beyond the physical aspects of their condition. The unifying theme of “abandoned in no man's land” serves as a metaphor for the participants' feelings of being thrust into unfamiliar and uncharted territory, where they must navigate and manage on their own. This experience brings forth complex emotions and considerations regarding life, death, treatment, and the future. Complementing this central theme, our study brings to light additional significant findings that enrich our understanding of the complex landscape patients must navigate while waiting for cardiac surgery. Particularly striking is the delicate balance these patients maintain between feeling imprisoned by their current condition and nurturing hopes of escape. Concurrently, they prepare for the upcoming fight against their illness, juxtaposed with experiences of vulnerability and reliance on social support. These intertwining themes paint a comprehensive picture of the patient journey, highlighting the breadth of their resilience and the depth of their challenges.

The term no man's land etymologically roots, denoting an indeterminate or ambiguous area without clear rules or authority, often depicted as a barren tract of land [42], that aptly captures the psychological and emotional limbo experienced by patients with IHD awaiting CABG surgery in this study. This metaphor resonates with the existential uncertainty, vulnerability, and fear reported in the literature, as patients navigate a period marked by suspended time and diminished control [9, 38–41]. According to van Manen, lived space (spatiality) is not just physical but also felt space that reflects the experience of lived time and lived body as a prereflective experience of space in which we find ourselves, which profoundly influences our emotional states [26]. Patients with IHD frequently report distressing physical symptoms [11, 38]. Drawing on van Manen's theoretical framework, our findings elucidate how these physical symptoms reflect those associated with heart disease, creating a scenario where the body seems to communicate in a familiar yet altered language. This confusion reflects a broader disruption in bodily integrity, as theorized by Benner, who argues that illness interrupts the taken-for-granted nature of the body, leading to a deep sense of loss of bodily integrity [43]. Van Manen further illuminates this phenomenon by suggesting that illness, forces patients to shift from a self-forgetful mode of being to a heightened awareness of the body. This shift alters not only the relationship with the body but also alters one's sense of time, priorities, experience of space, relations with others, and self-perception [44]. The body, once experienced as a seamless part of oneself, becomes “object-like” and experienced as encumbered, emphasizing the dissonance between one's physical state and identity [44]. These existential disruptions align with, Meleis' transitions theory [34], on health-illness transitions as more than just physical events, but involve an “inner reorientation and self-redefinition” to integrate change into one's life. Transition occurs when a person's reality is disrupted, necessitating a shift to construct a new reality [34]. According to our findings, this reorientation manifests as participants developing control over their uncertain situation through various coping strategies. Whether continuing daily life routines to keep stress at bay or engaging in preparatory activities focusing on practical concerns, all participants experienced a form of no man's land, a state of existential limbo leading to feelings of powerlessness and vulnerability. This resonates with previous research indicating the waiting period before CABG surgery as not only complex [39] but potentially distressing or traumatic [45]. Such experiences can significantly impair quality of life, particularly by diminishing both physical and social functioning [5].

The experience of waiting for CABG surgery is influenced not only by the nature of illness but also by individual patient characteristics and the patient`s subjective sense of time [24]. As our findings show, patients often feel suspended in a state of uncertainty, where time seems to stretch indefinitely and control over daily life diminishes. These experiences resonate with Charmaz's exploration of chronic illness, where altered perceptions of time and control contribute to a disrupted sense of the self [36]. She describes this as “lost time” and a “loss of control over time,” characterizing it as being “locked into a protracted limbo” [36]. Such experiences often lead to a profound loss of self, as patients witness their former self-images erode without the emergence of new ones that hold comparable value [36]. This sense of liminality was evident in our participants' narratives, particularly in terms of how patients feel sidelined in their own lives, disconnected from their diseased bodies, symbolized by their unreliable hearts. Yet, our findings also highlight a counterpoint; some participants manage to reclaim a sense of agency by actively reengaging with life. Through small acts of control, hope, and preparation, they began to reconstruct a sense of self and purpose. This reengagement reflects a dynamic process of adaptation, where patients move from passivity to participation, gradually restoring confidence and resilience in the face of uncertainty. Our findings illuminate how patients navigate a delicate balance between feeling imprisoned and hoping to escape through CABG surgery. Hope, inherently linked to human existence, is a dynamic and multidimensional phenomenon involving emotional, cognitive, motivational, social, and identity-related components. It serves as a consolation amid life's challenges [46]. In this context, CABG surgery emerges not only as a medical procedure but as a beacon of hope for regaining a functional life, which starkly contrasts with the existential threat posed by the wait. The metaphor of imprisonment captures the physical and psychological constraints imposed by ischemic heart limitations that disrupt daily life, diminish self-esteem, and evoke feelings of powerlessness, sadness, anger, and anxiety, impacting family and social relationships [40]. Participants describe a sense of “me yet not me,” reflecting a fractured identity and a state of existential limbo. Charmaz emphasizes that uncertainty about health can escalate to fear and dread, particularly when symptoms change. Such changes heighten individuals' awareness of time and prompt a fight against illness [36]. In our study, participants described the waiting period before CABG surgery not merely as a passive interval but as a space for envisioning recovery, a time to imagine their bodies regaining vitality and reclaiming the freedom to engage in everyday life. Expectations of recovery from the CABG surgery foster a sense of meaningfulness and coherence in their lives, echoing Antonovsky' s notion that perceiving challenges as meaningful can motivate individuals to invest in overcoming them [35].

Furthermore, our study reveals how patients awaiting CABG surgery navigate the experience of living with a changed body, describing their preparation as a “battle.” This metaphor reflects a disrupted relationship with the body, resonating with van Manen's phenomenological view of illness, where [44] disrupted relationship with the body is a characteristic of illness experiences, underscoring the need for a livable bodily relationship within nursing care. Moreover, these findings resonate with Antonovsky' s concept of SOC [35], which emphasizes the capacity to perceive life as comprehensible, manageable, and meaningful. Rather than succumbing to passivity, participants adopted their own strategies, such as dietary changes and increased physical activity, not just as a coping mechanism but as transformative actions that empowered them during the wait time. Some participants described creating both physical and mental space through daily walks, This aligns with research suggesting that patients waiting for cardiovascular surgery can develop a strong SOC through long-term engagement with their condition and the use of self-regulatory strategies [47]. From Antonovsky' s salutogenetic perspective, SOC serves as an inner strength that enables individuals to manage stress and maintain well-being, so a strong SOC may act as a protective factor, enabling patients to navigate the uncertainty of the waiting period with greater confidence.

In this study, patients express concern that friends may not fully comprehend the seriousness of their condition or offer the empathy they need, prompting them to process emotions alone or turn to healthcare professionals for support. This aligns with Kralik, who argues that individuals with chronic illnesses experience a shift in self-perception during transitional periods, adapting to a new reality and their world differently [48], as they adapt to changes rather than returning to a former state. Meleis et al. emphasize that effective transitions involve awareness of change, engagement with the transition process, and activities such as seeking support and redefining one's lifestyle and identity [34]. For participants in this study, the waiting period before CABG surgery represents a profound transitional phase, an existential no man's land marked by uncertainty and emotional distress. Recognizing this is essential for nurses, who play a pivotal role in supporting and advising patients individually through such disruptive experiences preoperatively [40, 48, 49]. To enhance patient support and preparedness before CABG surgery, findings indicate that healthcare professionals should acknowledge and respect patient`s individual values, cultural background, and decision-making process [50].

### 6.1. Methodological Considerations and Limitations

In applying phenomenological philosophy to this study, we adhered to methodological principles derived from van Manen's approach. Central to this approach is maintaining an open attitude throughout the research process, allowing the lived experience of participants to emerge authentically. The validity of phenomenological research findings depends on the extent to which they illuminate the meanings inherent in narrated lived experience, shedding light on the phenomenon under investigation. Our study aimed not to generalize but to uncover experiential structures essential to the phenomenon of waiting for CABG surgery, aiming for a depth that reveals universal human experiences regardless of context (van Manen). The purpose of phenomenological writing is to employ rigorous and nuanced language sensitive to the studied phenomenon to foster heuristic questioning, experiential richness, interpretive depth, and reflective rigor among readers. Readers engaging with phenomenological texts are thus invited to explore questions such as, What does it mean to experience waiting for? How do individuals with heart disease articulate the unique significance of waiting for CABG surgery? By engaging deeply with these questions, our study strives to provide a valid and comprehensive exploration of the multifaceted experiences patients have during this critical period. While the findings of our study are drawn from a specific context, they are presumed to possess good transferability to patients experiencing waiting for surgery in similar settings based on the universality of human experiences. One strength of the study is its robust philosophical and methodological underpinnings, which enable a deep exploration of the lived experience of patients waiting for CABG surgery. This approach provides rich, contextually situated insights that are essential for comprehending the manifold dimensions of waiting for during this phase of the illness and treatment trajectory. However, this study may be limited in its ability to fully capture the diversity of experiences within the patient population waiting for CABG surgery, including variations in socioeconomic status, cultural backgrounds, and comorbidities. The timing and setting of the interviews, conducted at the hospital the day before surgery, might have influenced them with a degree of formality or social desirability, potentially limiting the depth of responses. Another limitation of this study is that the first author (D.B.O.) is employed at the heart surgery unit where the participants were admitted for surgery. The participants were informed about this, and that the results would be anonymized and would not affect their treatment. Nevertheless, this may have influenced the interview situation. Another limitation of this study concerns the duration of the interviews, which ranged from 12 to 42 min, with an average of 27 min. While some interviews allowed for in-depth exploration of participants' lived experiences, others, particularly those on the shorter end of the spectrum, may have constrained the richness and complexity of the data. Interviews lasting only 12 min may not have provided sufficient time for participants to fully articulate their perspectives, potentially limiting the depth of insight gained. Despite these limitations, the study offers valuable insights into a critical and often overlooked phase of the cardiac care journey. The findings contribute to a deeper understanding of how patients interpret and endure the uncertainty of waiting and invite further exploration of this emotionally and existentially charged experience. Empirical insights drawn from lived experience are characterized by their recognizability and potential for transferability, supporting their relevance across diverse contexts and settings.

## 7. Conclusion

The phenomena this study uncovered emphasize the complexities inherent to the patient's lived experience of waiting for CABG surgery, revealing that waiting is not merely a temporal delay but a deeply existential experience. Unlike previous research, it found that waiting for surgery is an existential experience marked by emotional vulnerability, loss of bodily integrity, and a sense of abandonment. The metaphor “abandoned in no man`s land” encapsulates the liminal space patients occupy, situated between the states of illness and recovery, self and others, oscillating between hope and despair, with emotions that are often silently endured and hidden from others. Moreover, this study emphasizes that waiting for CABG surgery is an active, meaning-making phase formed by patient's individual coping strategies and SOC. During this transitional period, patient's often experience emotional solitude, navigating their inner thoughts and fears in silence, as they strive to integrate change or construct a new reality into their lives. For nurses, attuning with concern and awareness to these unspoken dimensions of patient's experiences when waiting for CABG surgery is essential and underscores the need for a holistic nursing practice that addresses both the physical and existential challenges patients experience in this period. Deepening our understanding of the waiting experience as a serious psychological and existential transition enables future research to inform the development of a more compassionate and person-centered nursing practice to enhance preoperative support within cardiac nursing practice. Focusing on patient's needs, challenges and coping strategies are significant when developing targeted interventions. Understanding and integrating these aspects into prehabilitation programs for patients waiting for CABG surgery help ensure they feel supported, empowered, and prepared during their wait, ultimately enhancing surgical outcomes and overall quality of life.

## Data Availability

The data that support the findings of this study are available from the corresponding author upon reasonable request.

## Supporting Information

The SRQR checklist [31].

## References

[1] Tadayon S., Wickramasinghe K., Townsend N. (2019). Examining Trends in Cardiovascular Disease Mortality Across Europe: How Does the Introduction of a New European Standard Population Affect the Description of the Relative Burden of Cardiovascular Disease?. *Population Health Metrics*.

[2] Timmis A., Vardas P., Townsend N. (2022). European Society of Cardiology: Cardiovascular Disease Statistics 2021. *European Heart Journal*.

[3] Schmidt-RioValle J., Abu Ejheisheh M., Membrive-Jiménez M. J. (2020). Quality of Life After Coronary Artery Bypass Surgery: A Systematic Review and Meta-Analysis. *International Journal of Environmental Research and Public Health*.

[4] Sousa-Uva M., Neumann F. J., Ahlsson A. (2019). 2018 ESC/EACTS Guidelines on Myocardial Revascularization. *European Journal of Cardio-Thoracic Surgery*.

[5] Sampalis J., Boukas S., Liberman M., Reid T., Dupuis G. (2001). Impact of Waiting Time on the Quality of Life of Patients Awaiting Coronary Artery Bypass Grafting. *Canadian Medical Association Journal: Canadian Medical Association Journal=Journal de l’Association Medicale Canadienne*.

[6] Waite I., Deshpande R., Baghai M., Massey T., Wendler O., Greenwood S. (2017). Home-Based Preoperative Rehabilitation (Prehab) to Improve Physical Function and Reduce Hospital Length of Stay for Frail Patients Undergoing Coronary Artery Bypass Graft and Valve Surgery. *Journal of Cardiothoracic Surgery*.

[7] Arthur H. M., Daniels C., McKelvie R., Hirsh J., Rush B. (2000). Effect of a Preoperative Intervention on Preoperative and Postoperative Outcomes in low-risk Patients Awaiting Elective Coronary Artery Bypass Graft Surgery. A Randomized, Controlled Trial. *Annals of Internal Medicine*.

[8] Mooney M., Fitzsimons D., Richardson G. (2007). No More Couch-Potato! Patients’ Experiences of a Pre-Operative Programme of Cardiac Rehabilitation for Those Awaiting Coronary Artery Bypass Surgery. *European Journal of Cardiovascular Nursing*.

[9] Feuchtinger J., Burbaum C., Heilmann C. (2014). Anxiety and Fear in Patients With Short Waiting Times Before Coronary Artery Bypass Surgery–A Qualitative Study. *Journal of Clinical Nursing*.

[10] Fitzsimons D., Parahoo K., Richardson S. G., Stringer M. (2003). Patient Anxiety While on a Waiting List for Coronary Artery Bypass Surgery: A Qualitative and Quantitative Analysis. *Heart & Lung*.

[11] Karlsson A.-K., Lidell E., Johansson M. (2008). Depressed Mood Over Time After Open Heart Surgery Impacts Patient Well-Being: A Combined Study. *European Journal of Cardiovascular Nursing*.

[12] Carli F., Scheede-Bergdahl C. (2015). Prehabilitation to Enhance Perioperative Care. *Anesthesiology Clinics*.

[13] McCann M., Stamp N., Ngui A., Litton E. (2019). Cardiac Prehabilitation. *Journal of Cardiothoracic and Vascular Anesthesia*.

[14] Arora R. C., Brown C. H., Sanjanwala R. M., McKelvie R. (2018). NEW Prehabilitation: A 3-Way Approach to Improve Postoperative Survival and Health-Related Quality of Life in Cardiac Surgery Patients. *Canadian Journal of Cardiology*.

[15] Marmelo F., Rocha V., Gonçalves D. (2018). The Impact of Prehabilitation on Post-Surgical Complications in Patients Undergoing Non-urgent Cardiovascular Surgical Intervention: Systematic Review and Meta-Analysis. *European Journal of Preventive Cardiology*.

[16] Sawatzky J. A. V., Kehler D. S., Ready A. E. (2014). Prehabilitation Program for Elective Coronary Artery Bypass Graft Surgery Patients: A Pilot Randomized Controlled Study. *Clinical Rehabilitation*.

[17] Carli F. (2022). Prehabilitation: Introduction. *Seminars in Oncology Nursing*.

[18] Scheede‐Bergdahl C., Minnella E. M., Carli F. (2019). Multi-Modal Prehabilitation: Addressing the Why, When, What, How, Who and Where Next?. *Anaesthesia*.

[19] Yamikan H., Ahiskali G. N., Demirel A., Kütükcü E. C. (2025). The Effects of Exercise-Based Prehabilitation in Patients Undergoing Coronary Artery Bypass Grafting Surgery: A Systematic Review of Randomized Controlled Trials. *Heart & Lung*.

[20] Steinmetz C., Heinemann S., Kutschka I. (2023). Prehabilitation in Older Patients Prior to Elective Cardiac Procedures (PRECOVERY): Study Protocol of a Multicenter Randomized Controlled Trial. *Trials*.

[21] Ferreira V., Agnihotram R. V., Bergdahl A. (2018). Maximizing Patient Adherence to Prehabilitation: What Do the Patients Say?. *Supportive Care in Cancer*.

[22] Karlsson A.-K., Johansson M., Lidell E. (2005). Fragility—The Price of Renewed Life. Patients Experiences of Open Heart Surgery. *European Journal of Cardiovascular Nursing*.

[23] Bargnes V., Davidson S., Talbot L., Jin Z., Poppers J., Bergese S. D. (2024). Start Strong, Finish Strong: A Review of Prehabilitation in Cardiac Surgery. *Life*.

[24] Carr T., Teucher U., Mann J., Casson A. G. (2009). Waiting for Surgery From the Patient Perspective. *Psychology Research and Behavior Management*.

[25] Olsen D. B., Pedersen P. U., Noergaard M. W. (2023). Prehabilitation Before Elective Coronary Artery Bypass Grafting Surgery: A Scoping Review. *JBI Evidence Synthesis*.

[26] van Manen M. (2006). *Researching Lived Experience: Human Science for an Action Sensitive Pedagogy*.

[27] van Manen M. (2023). *Phenomenology of Practice: Meaning-Giving Methods in Phenomenological Research and Writing*.

[28] van Manen M. (2017). Phenomenology in its Original Sense. *Qualitative Health Research*.

[29] van Manen M. A. (2014). On Ethical (In)Decisions Experienced by Parents of Infants in Neonatal Intensive Care. *Qualitative Health Research*.

[30] van Manen M., van Manen M. (2021). Doing Phenomenological Research and Writing. *Qualitative Health Research*.

[31] O’Brien B. C., Harris I. B., Beckman T. J., Reed D. A., Cook D. A. (2014). Standards for Reporting Qualitative Research. *Academic Medicine*.

[32] van Manen M., van M. M. (2021). *Classic Writings for Phenomenology of Practice*.

[33] Churchill S. D. (2012). Teaching Phenomenology by Way of “Secondperson Perspectivity” (From My Thirty Years at the University of Dallas). *Indo-Pacific Journal of Phenomenology*.

[34] Meleis A. I., Sawyer L. M., Im E.-O., Hilfinger Messias D. K., Schumacher K. (2000). Experiencing Transitions: An Emerging Middle-Range Theory. *Advances in Nursing Science*.

[35] Antonovsky A. (1979). Health, Stress, and Coping: New Perspective on Mental and Physical Well-Being.

[36] Charmaz K. (1991). God Days, Bad Days, the Self in Chronic Illness and Time 1991.

[37] (2024). World Medical Association Declaration of Helsinki. *Journal of the American Medical Association*.

[38] Fitzsimons D., Parahoo K., Stringer M. (2000). Waiting for Coronary Artery Bypass Surgery: a Qualitative Analysis. *Journal of Advanced Nursing*.

[39] Kathania D. S., Singh N. V., Kaur S., Kumar R. (2021). Patients Perception About Coronary Artery Bypass Graft (CABG) Surgery During Waiting Period: A Phenomenological Study. *Nursing & Midwifery Research Journal*.

[40] McCormick K. M., McClement S., Naimark B. J. (2005). A Qualitative Analysis of the Experience of Uncertainty While Awaiting Coronary Artery Bypass Surgery. *Canadian Journal of Cardiovascular Nursing=Journal Canadien En Soins Infirmiers Cardio-Vasculaires*.

[41] McCormick K. M., Naimark B. J., Tate R. B. (2006). Uncertainty, Symptom Distress, Anxiety, and Functional Status in Patients Awaiting Coronary Artery Bypass Surgery. *Heart & Lung*.

[42] (2023). Cambridge Dictionary. https://Dictionary.Cambridge.Org/.

[43] Benner P. (1989). *The Primacy of Caring. Stress and Coping in Health and Illness*.

[44] van Manen M. (1998). Modalities of Body Experience in Illness and Health. *Qualitative Health Research*.

[45] Ivarsson B., Larsson S., Sjöberg T. (2004). Patients’ Experiences of Support While Waiting for Cardiac Surgery. A Critical Incident Technique Analysis. *European Journal of Cardiovascular Nursing*.

[46] Antunes M., Laranjeira C., Querido A., Charepe Z. (2023). What Do We Know About Hope in Nursing Care?: A Synthesis of Concept Analysis Studies. *Health Care*.

[47] Matsushita T., Ohki T., Hamajima M., Matsushima E. (2007). Sense of Coherence Among Patients With Cardiovascular Disease and Cancer Undergoing Surgery. *Holistic Nursing Practice*.

[48] Kralik D., Visentin K., van Loon A. (2006). Transition: A Literature Review. *Journal of Advanced Nursing*.

[49] Aase Schaufel M., Nordrehaug J. E., Malterud K. (2011). Hope in Action-Facing Cardiac Death: A Qualitative Study of Patients With Life-Threatening Disease. *International Journal of Qualitative Studies on Health and Well-Being*.

[50] Ebrahimi S., Tehrani T. H., Azizi A., Vahedparast H., Sadeghian E. (2025). Challenges Faced by Cardiac Patients Prior to Coronary Artery Bypass Grafting: A Qualitative Study. *BMC Cardiovascular Disorders*.

